# Supplementary material for: Borneol promotes autophagic degradation of HIF-1α and enhances chemotherapy sensitivity in malignant glioma
Source: PeerJ. 2024 Jan 3;12:e16691. doi: 10.7717/peerj.16691 (PMC10771087; doi:10.7717/peerj.16691)
Supplement: Supplemental Information 1 [file peerj-12-16691-s001.pdf]

CCK8

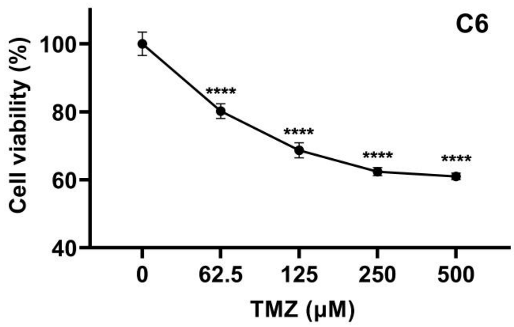
C6
Cell viability (%)
100
80
60
40
****
0
62.5
125
250
500
TMZ (μM)

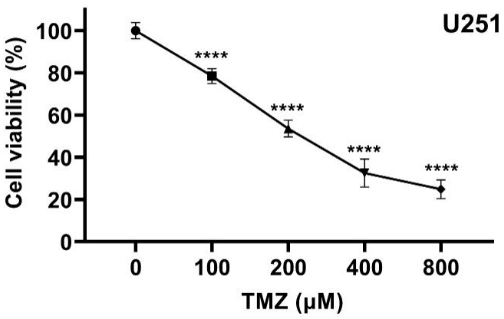
U251
Cell viability (%)
100
80
60
40
20
0
****
0
100
200
400
800
TMZ (μM)

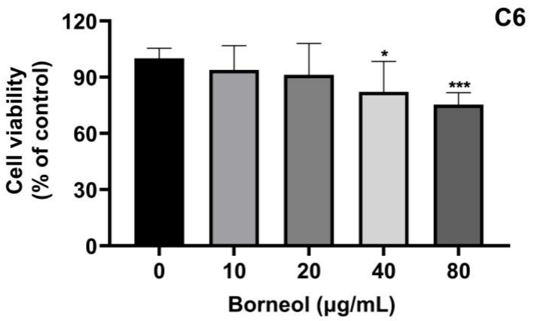
C6
Cell viability
(% of control)
120
90
60
30
0
*
***
0
10
20
40
80
Borneol (μg/mL)

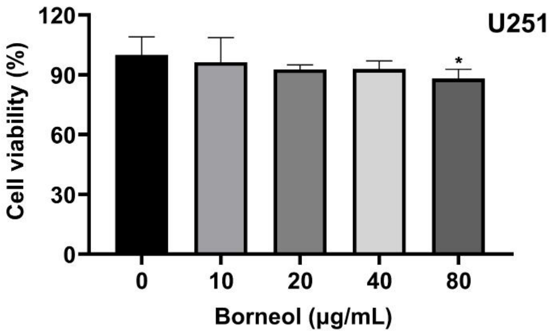
U251
Cell viability (%)
120
90
60
30
0
*
0
10
20
40
80
Borneol (μg/mL)

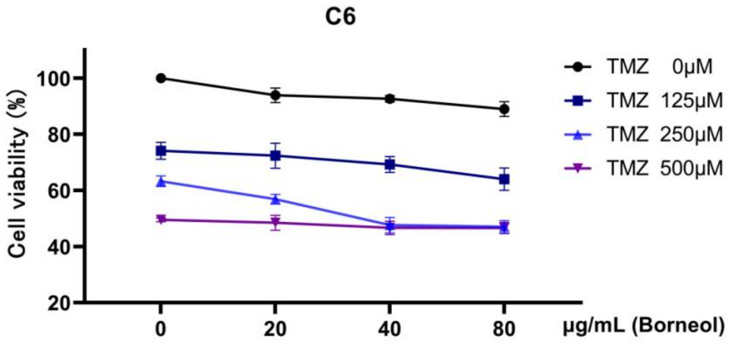
C6
Cell viability (%)
100
80
60
40
20
TMZ 0μM
TMZ 125μM
TMZ 250μM
TMZ 500μM
0
20
40
80
μg/mL (Borneol)

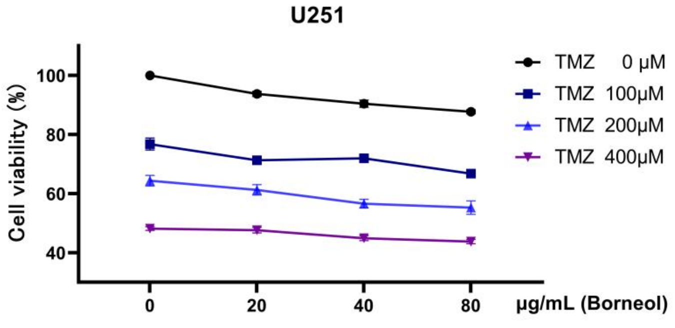
U251
Cell viability (%)
100
80
60
40
TMZ 0 μM
TMZ 100μM
TMZ 200μM
TMZ 400μM
0
20
40
80
μg/mL (Borneol)

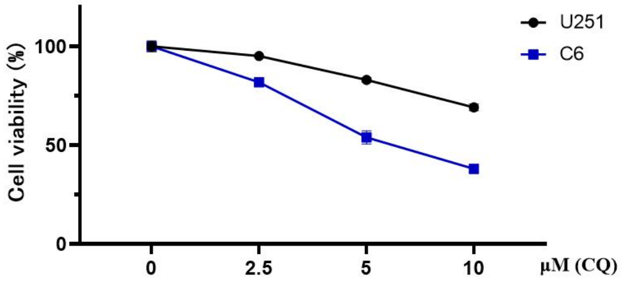
Cell viability (%)
100
50
0
U251
C6
0
2.5
5
10
μM (CQ)

Clonogenic assay

C6

Control | 250μM TMZ | 500μM TMZ

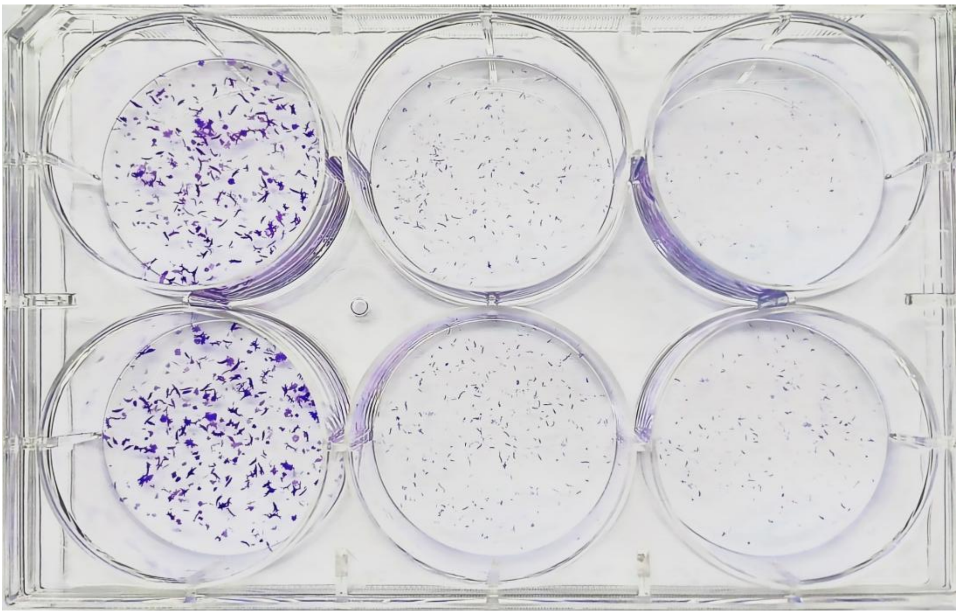

40μg/ml Borneol | 250μM TMZ+ 40μg/ml Borneol | 250μM TMZ+ 80μg/ml Borneol

U251

Control | 200μM TMZ | 400μM TMZ

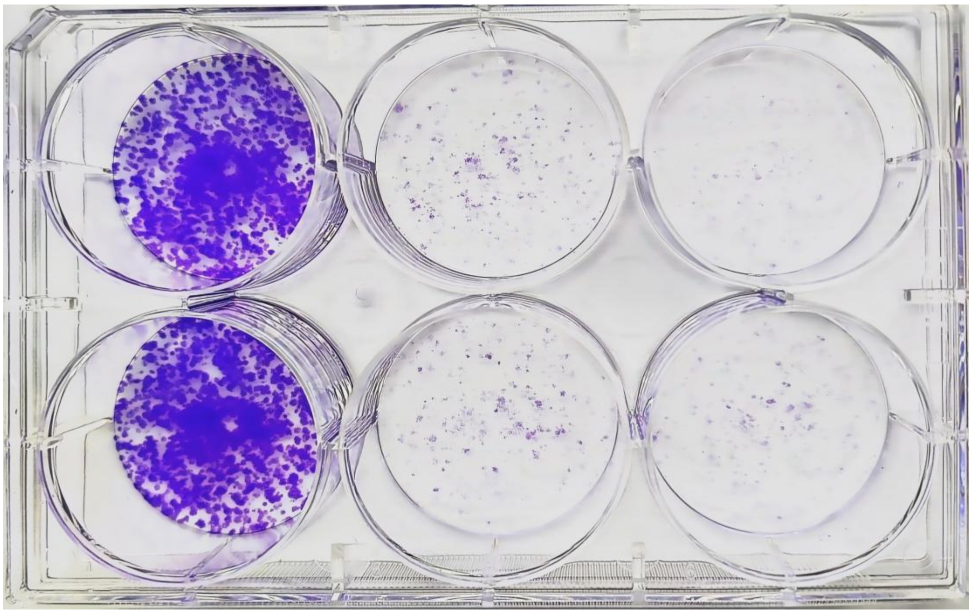

40μg/ml Borneol | 200μM TMZ+ 40μg/ml Borneol | 200μM TMZ+ 80μg/ml Borneol

HE

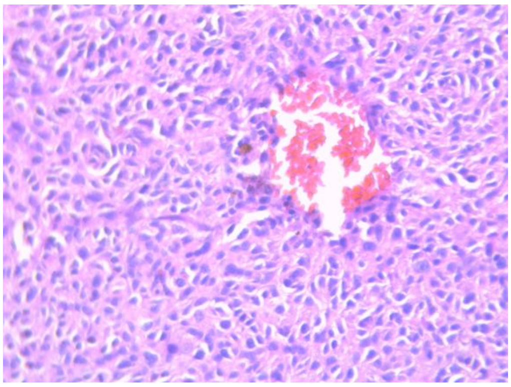

Control

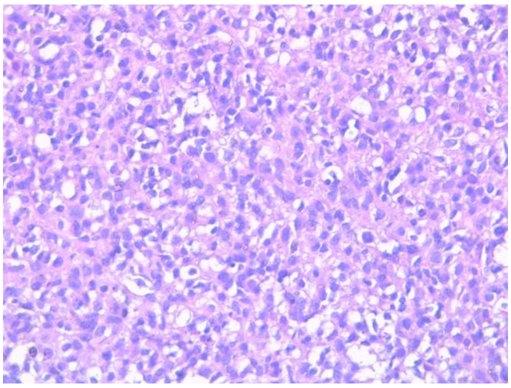

Borneol

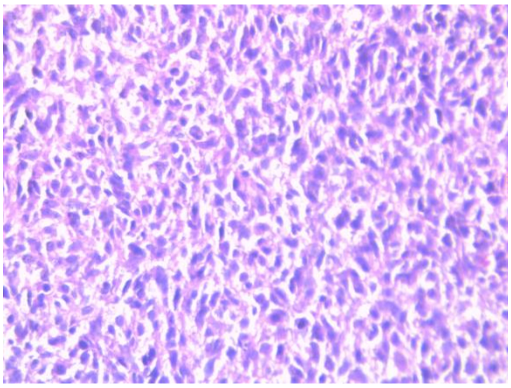

TMZ

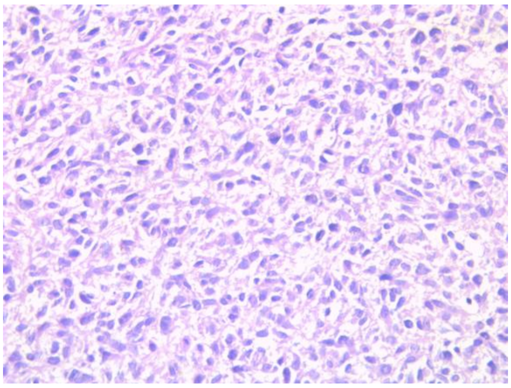

TMZ+Borneol

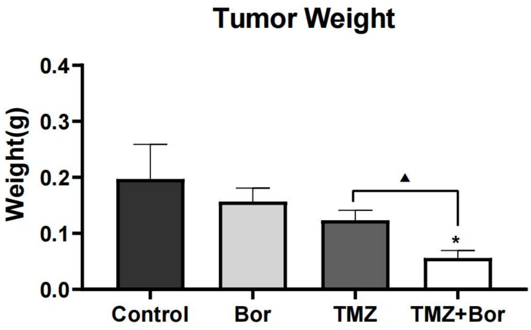

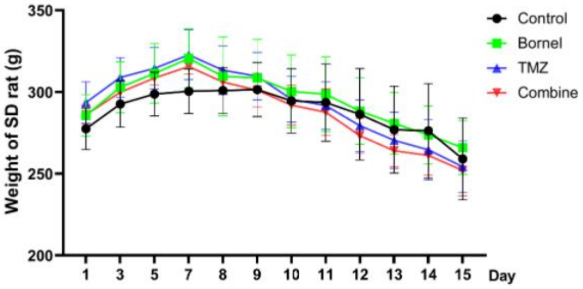

TEM

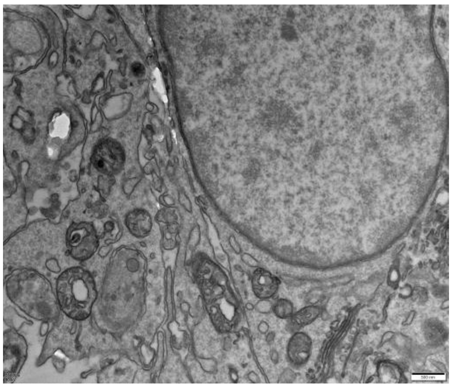

Control

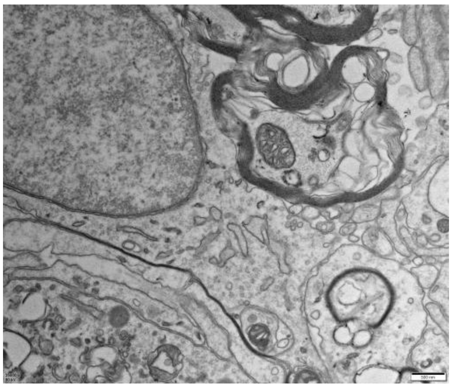

Borneol

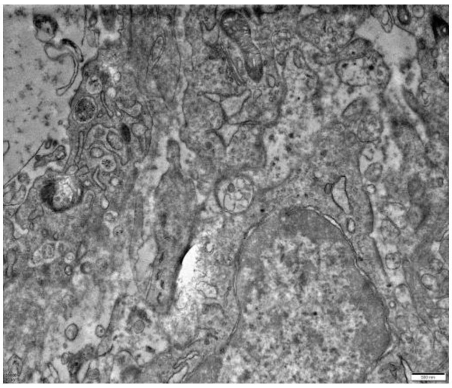

TMZ

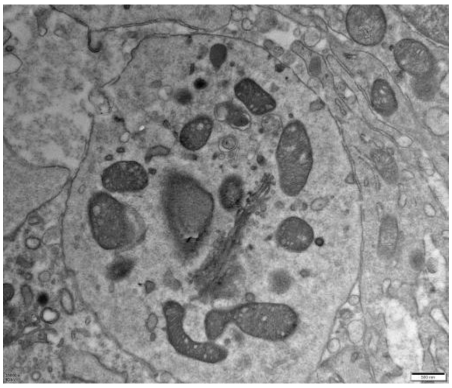

TMZ+Borneol

IHC

HIF-1α

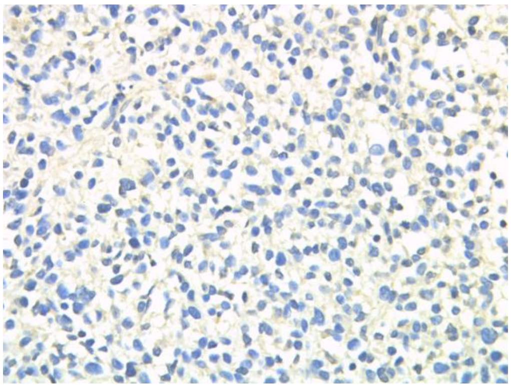

Control

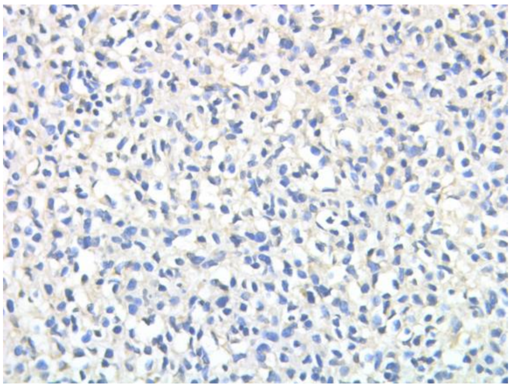

Borneol

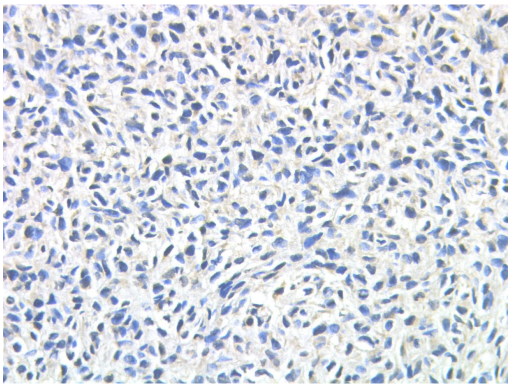

TMZ

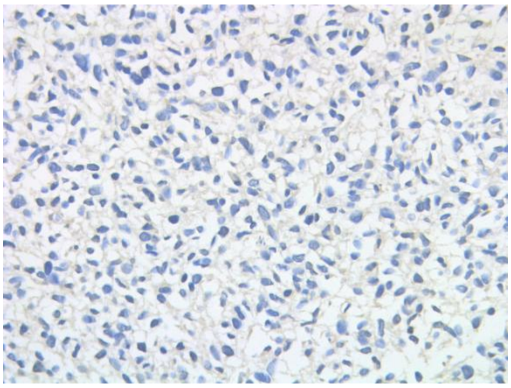

TMZ+Borneol

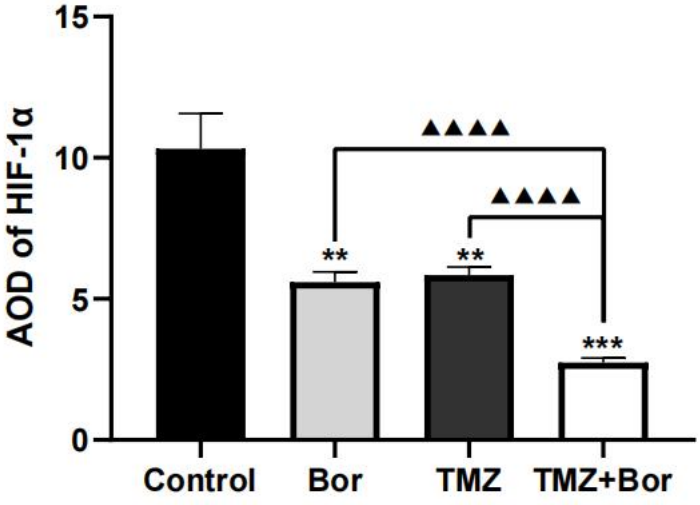

IHC

Beclin-1

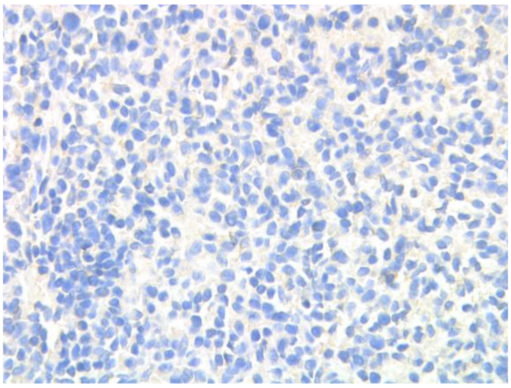

Control

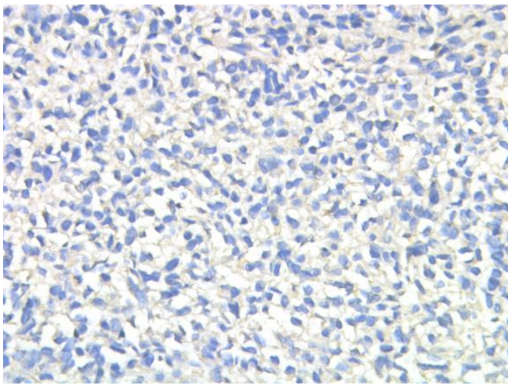

Borneol

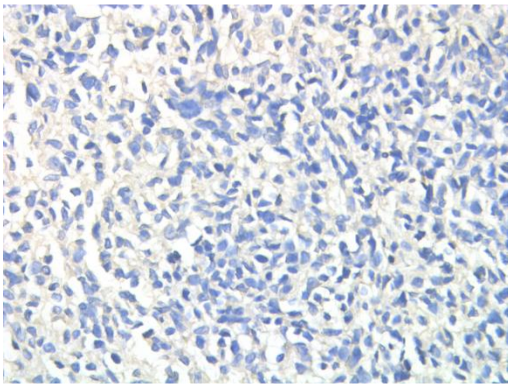

TMZ

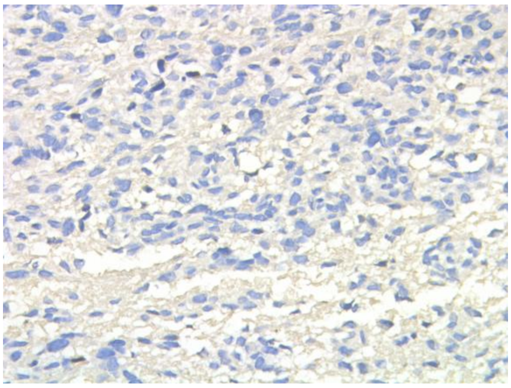

TMZ+Borneol

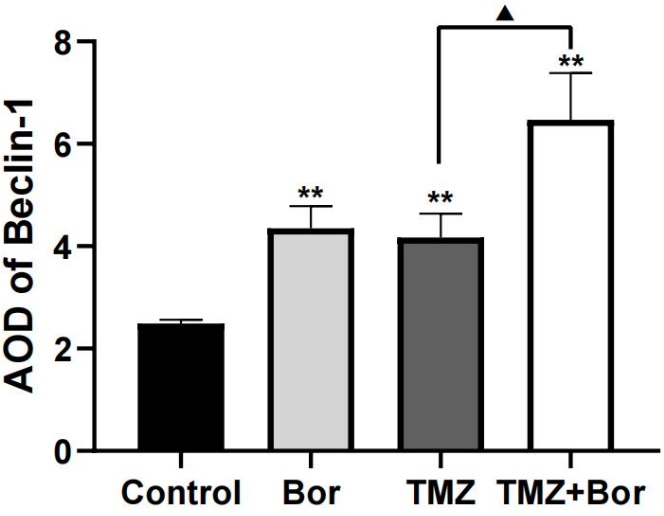

IHC

LC3A/B

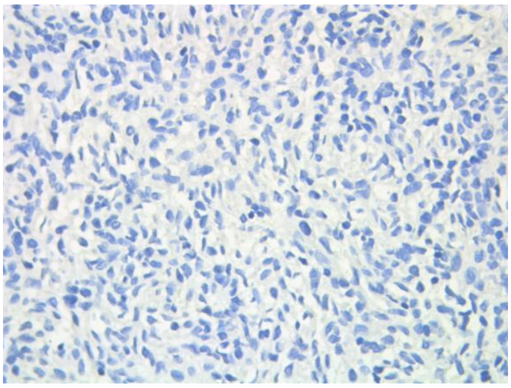

Control

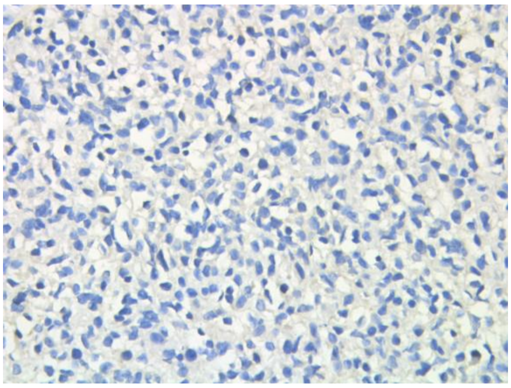

Borneol

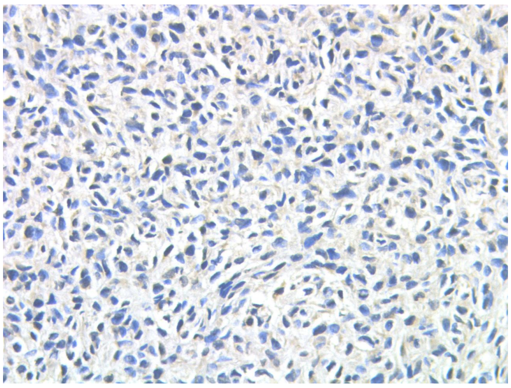

TMZ

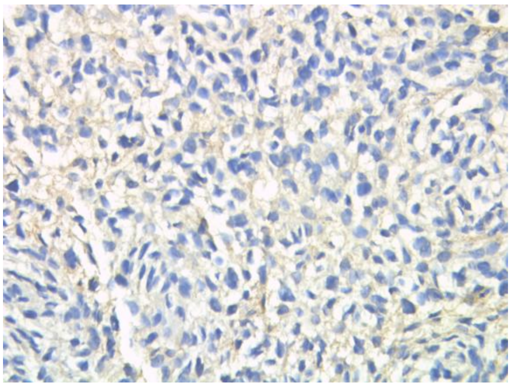

TMZ+Borneol

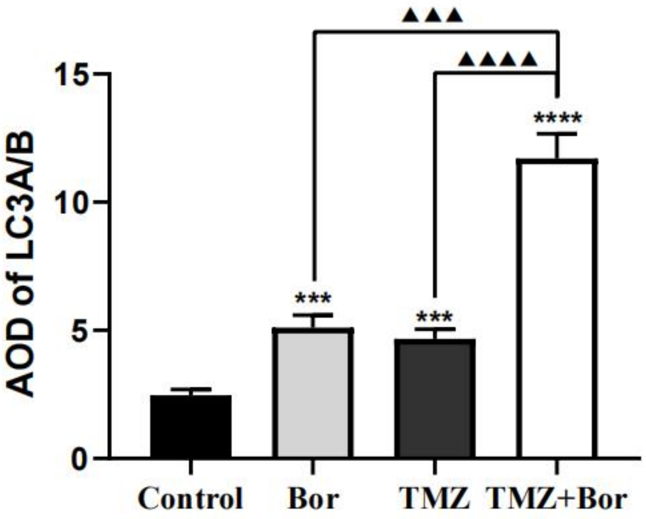

Apoptosis

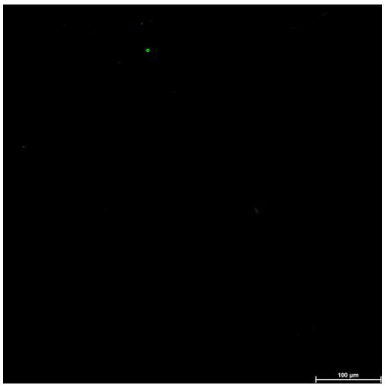
Control

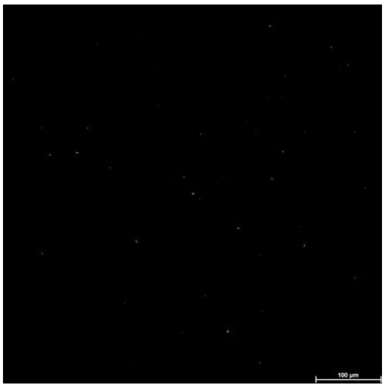
Borneol

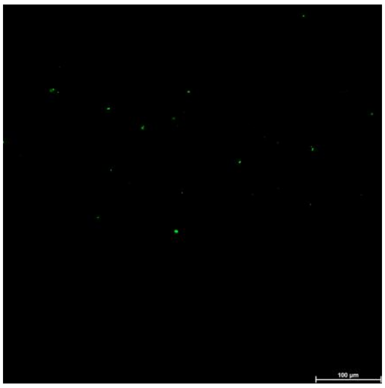
TMZ

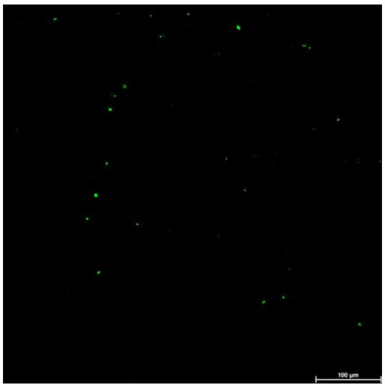
TMZ+Borneol
