# Supplementary material for: Borneol promotes autophagic degradation of HIF-1α and enhances chemotherapy sensitivity in malignant glioma
Source: PeerJ. 2024 Jan 3;12:e16691. doi: 10.7717/peerj.16691 (PMC10771087; doi:10.7717/peerj.16691)
Supplement: Supplemental Information 2 [file peerj-12-16691-s002.docx]

**C6**

Con 40B 80B 250T

250T

+40B

250T

+80B


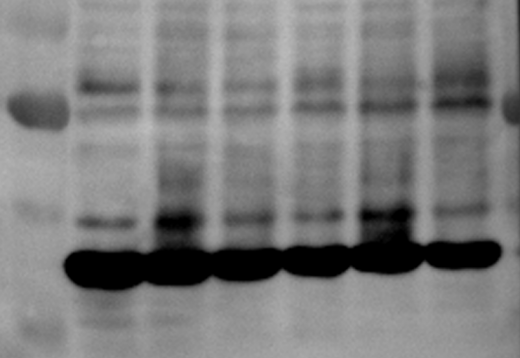

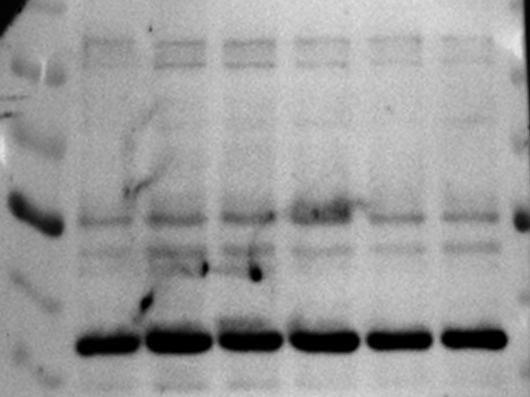


**Repeat 1**


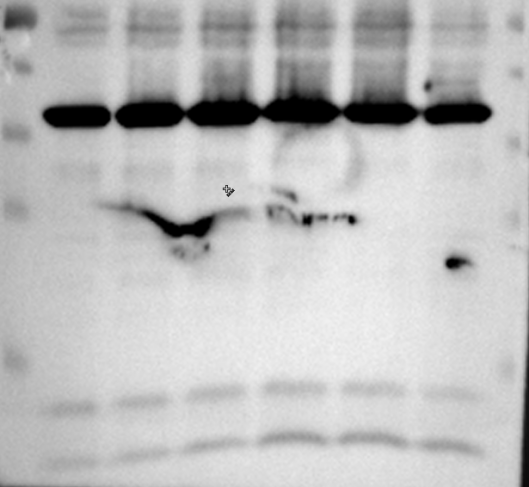


HIF-1α 120kDa

GAPDH 36kDa

GAPDH 36kDa

Beclin 1 52kDa

LC3Ⅰ 16kDa

LC3Ⅱ 14kDa

40

35

130

70

100

40

55

35

55

LC3Ⅱ

LC3Ⅰ

Beclin 1

GAPDH

HIF-1αα

GAPDH

15

35

40

25

GAPDH

**Repeat 2**


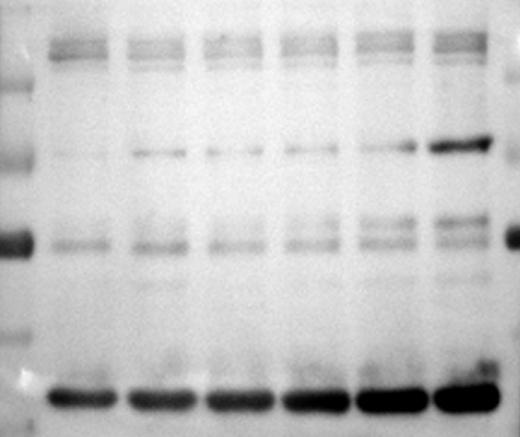

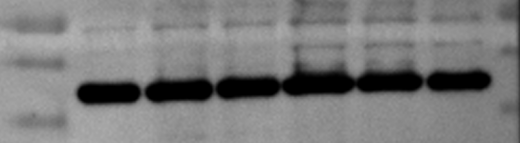


**Repeat 3**

130

70

100

40

55

35

Con 40B 80B 250T

250T

+80B

250T

+40B

Con 40B 80B 250T

250T

+80B

250T

+40B


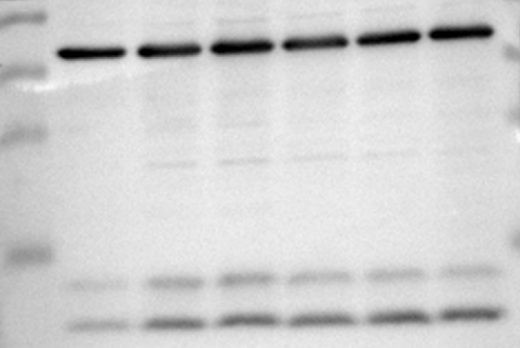


70

40

55

35

15

55

35

40

25


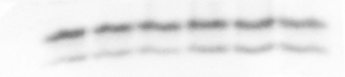

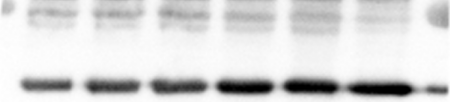


130

55

Beclin 1

GAPDH

HIF-1αα

LC3Ⅱ

LC3Ⅰ

GAPDH

GAPDH 36kDa

35

40

100


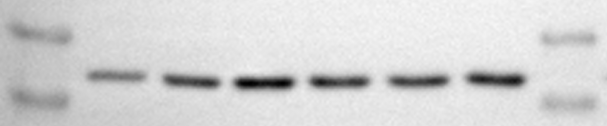

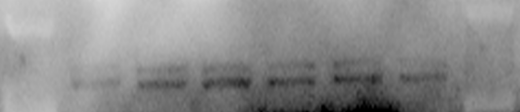


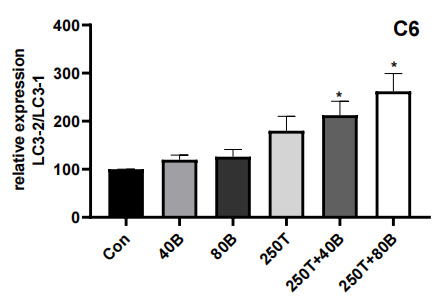

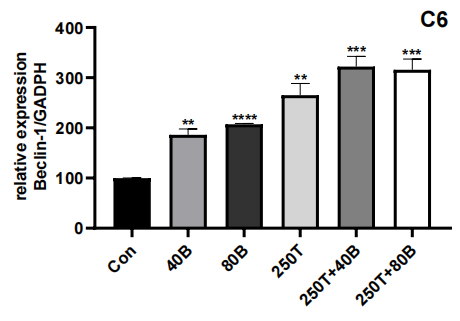

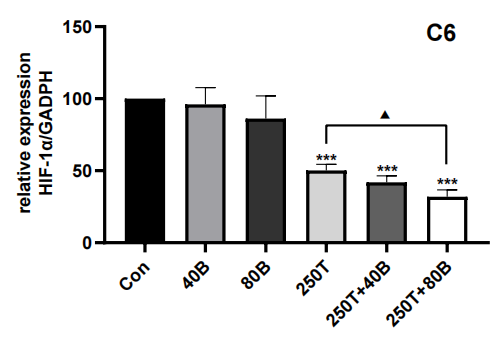


**U251**

GAPDH 36kDa

HIF-1α 120kDa

Beclin 1 52kDa


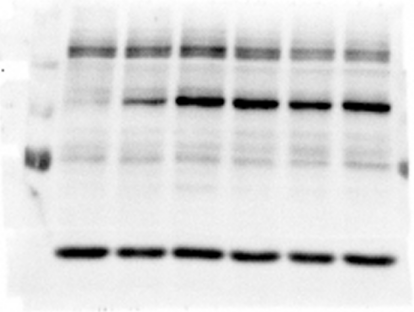


Con 40B 80B 200T

200T

+80B

200T

+40B

130

70

100

40

55

**Repeat 1**

GAPDH 36kDa

55


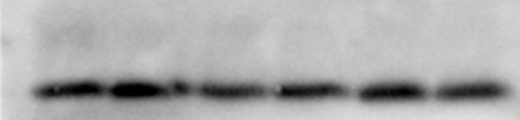

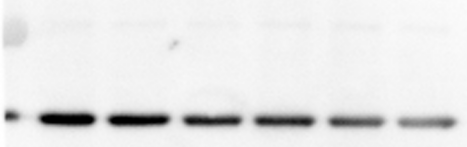


LC3Ⅱ 16kDa 16kDa

LC3Ⅰ 14kDa

15


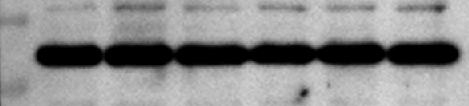

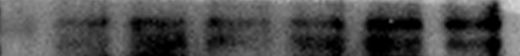

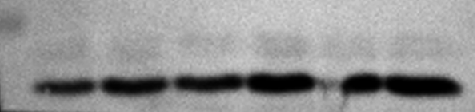

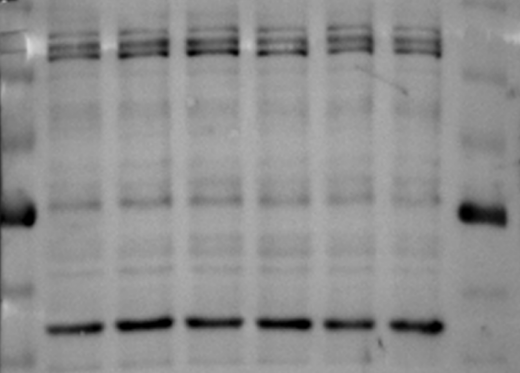


40

35

130

70

100

40

55

35

55

Beclin 1

GAPDH

HIF-1αα

GAPDH

Con 40B 80B 200T

200T

+80B

200T

+40B

**Repeat 2**

LC3Ⅱ

LC3Ⅰ

15


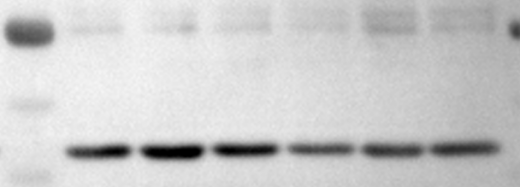

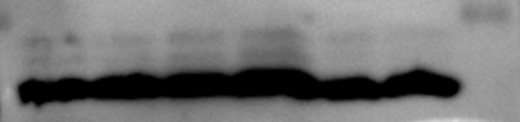

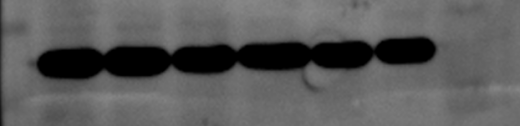

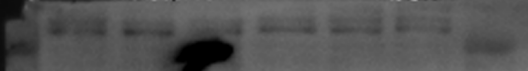


Con 40B 80B 200T

200T

+80B

200T

+40B

130

100

40

55

35

40

35

LC3Ⅱ

LC3Ⅰ

15

Beclin 1

GAPDH

HIF-1αα

GAPDH

**Repeat 3**


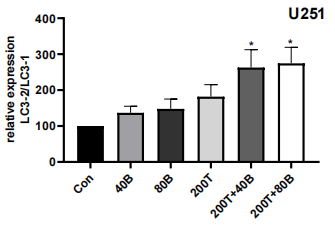

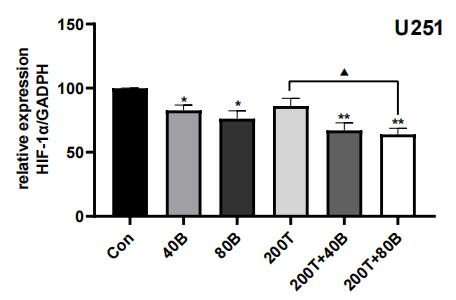


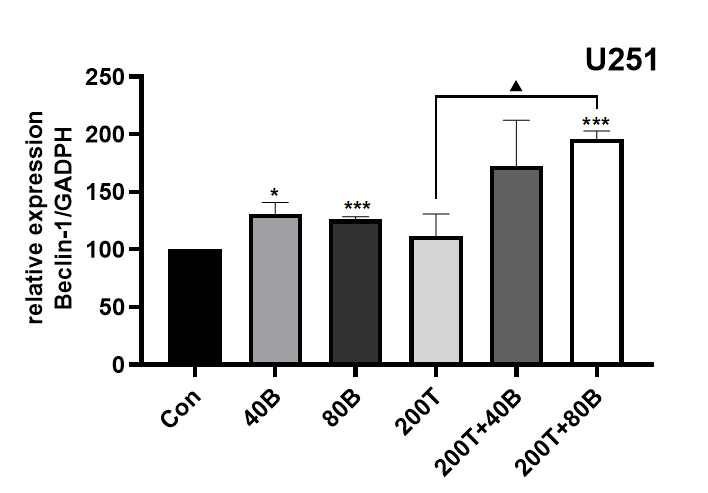


130

HIF-1α 120kDa

100

Con CQ 40B

CQ+40B


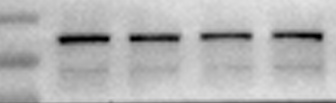


**Repeat 1**

40


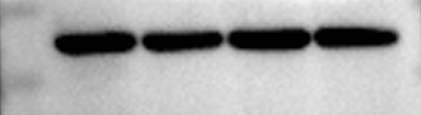


GAPDH 37kDa

35

130

100

40

35

GAPDH

HIF-1αα

**Repeat 2**

Con CQ 40B

CQ+40B


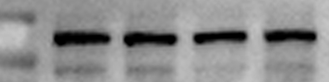

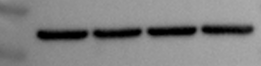


**Repeat 3**

130

100

HIF-1αα


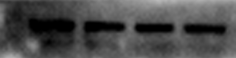


GAPDH

35

40


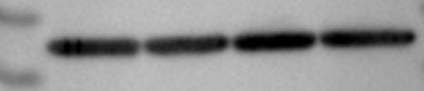


Con CQ 40B

CQ+40B

**U251**


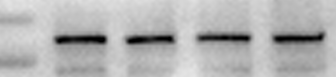

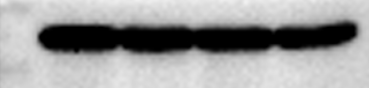


Con CQ 40B

CQ+40B

**Repeat 4**

40

GAPDH 37kDa

35

100

130

HIF-1α 120kDa


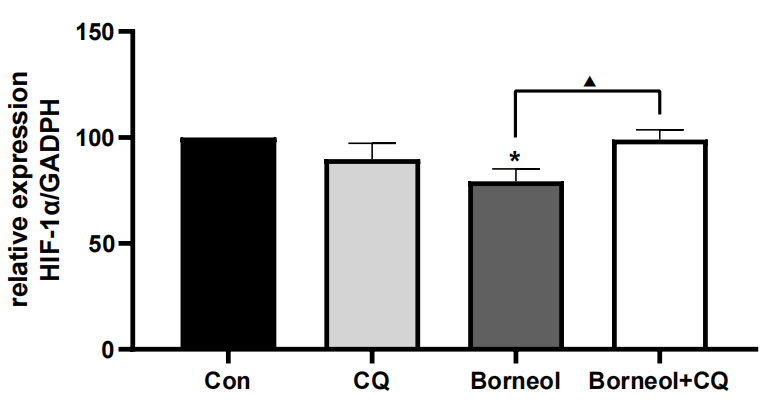


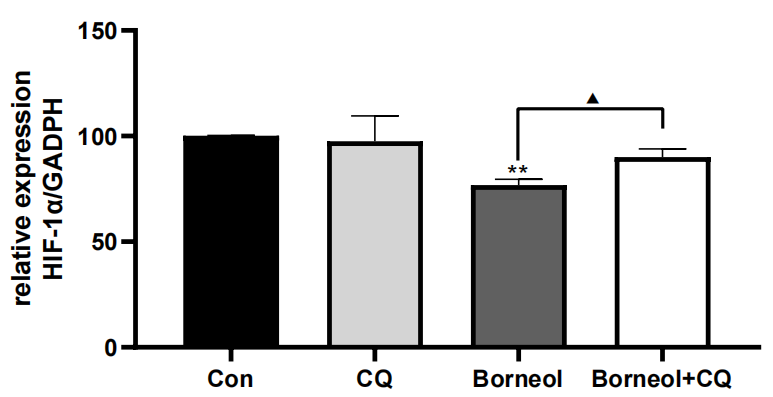

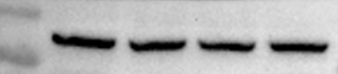

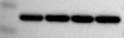

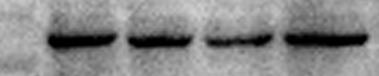

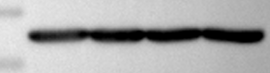

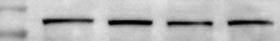


**Repeat 3**

130

100

HIF-1αα

GAPDH

35

40

Con CQ 40B

CQ+40B

130

100

40

35

GAPDH

HIF-1αα

**Repeat 2**

Con CQ 40B

CQ+40B

**Repeat 1**


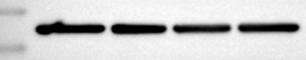


40

GAPDH 37kDa

35

130

HIF-1α 120kDa

100

**C6**

Con CQ 40B

CQ+40B
